# Moss Mediates the Influence of Shrub Species on Soil Properties and Processes in Alpine Tundra

**DOI:** 10.1371/journal.pone.0164143

**Published:** 2016-10-19

**Authors:** C. Guillermo Bueno, Scott N. Williamson, Isabel C. Barrio, Ágústa Helgadóttir, David S. HiK

**Affiliations:** 1 Institute of Ecology and Earth Sciences, Department of Botany, University of Tartu, Lai 40, Tartu, 51005, Estonia; 2 Pyrenean Institute of Ecology (CSIC), Avda. Nuestra Señora de la Victoria s/n, 22700, Jaca, Spain; 3 Department of Biological Sciences, University of Alberta, Biological Sciences Building, T6G 2E9, Edmonton, Canada; 4 Department of Life and Environmental Sciences, University of Iceland, Sturlugata 7, 101, Reykjavik, Iceland; 5 Soil Conservation Service of Iceland, Gunnarsholt, 851, Hella, Iceland; Portland State University, UNITED STATES

## Abstract

In tundra ecosystems, bryophytes influence soil processes directly and indirectly through interactions with overstory shrub species. We experimentally manipulated moss cover and measured seasonal soil properties and processes under two species of deciduous shrubs with contrasting canopy structures, *Salix planifolia pulchra* and *Betula glandulosa-nana* complex. Soil properties (seasonal temperature, moisture and C:N ratios) and processes (seasonal litter decomposition and soil respiration) were measured over twelve months. Shrub species identity had the largest influence on summer soil temperatures and soil respiration rates, which were higher under *Salix* canopies. Mosses were associated with lower soil moisture irrespective of shrub identity, but modulated the effects of shrubs on winter soil temperatures and soil C:N ratios so that moss cover reduced differences in soil winter temperatures between shrub species and reduced C:N ratios under *Betula* but not under *Salix* canopies. Our results suggest a central role of mosses in mediating soil properties and processes, with their influence depending on shrub species identity. Such species-dependent effects need to be accounted for when forecasting vegetation dynamics under ongoing environmental changes.

## Introduction

Many shrub species are expanding in northern and alpine tundra. Shrubification of the tundra biome has been attributed to amelioration of factors that limit shrub growth associated with both climate change and herbivory [1–4]. However, shrub expansion is sensitive to variation in local conditions, with precipitation and local disturbance often having a greater influence than temperatures [5]. Further, the decline of traditional husbandry in some alpine and arctic regions has reduced grazing pressure on shrubs, contributing to their rapid expansion [3, 6–8].

The mechanisms facilitating shrubification are not well understood. Increases in shrub abundance may promote or inhibit further shrub expansion through their effects on nutrient cycling. For example, taller shrub canopies could trap more snow in winter [9, 10]; the insulating snow layer could then maintain relatively warmer soils in winter and increase soil moisture in summer, promoting conditions for litter decomposition [11, 12] and thus increasing N availability [13]. In some circumstances this may promote further growth and expansion of fast growing shrubs [14, 15], although other studies have found no effect of snow accumulation on soil decomposition rates [12, 16]. Warming can accelerate soil nutrient cycling by speeding up growth rates and increasing the production of less recalcitrant leaf litter with lower C:N ratios [12, 17, 18]. While this may increase N availability in soils and subsequent shrub growth and expansion [4], there will also be a concomitant accumulation of woody litter. This woody litter in the soil may slow subsequent decomposition, reduce available soil N availability, retarding shrub growth [17, 19]. Since the variability in soil C and nutrients in relation to the rapid shrub expansion is not fully accounted for, other processes and actors are likely modulating these changes [11, 12, 16, 17, 20].

Mosses can play a significant role in controlling belowground processes in boreal forest [21] and High Arctic environments by controlling abiotic soil conditions [22, 23]. Mosses have low thermal conductivity and are efficient insulators, preventing water infiltration into soil and soil moisture loss to evapotranspiration [21–24]. Mosses also contribute to N and C cycles by fixing atmospheric N through symbiotic relationships with cyanobacteria [25], and by producing recalcitrant litter [19, 26, 27]. While fixed N can be quickly released into the soil upon drying and rewetting moss tissue [28, 29], recalcitrant moss litter traps C, slowing decomposition and reducing soil respiration rates[21, 22]. In this context, the effects of shade-tolerant mosses growing under shrubs in alpine and arctic environments are particularly relevant [24]

Mosses influence nutrient cycling, ecosystem dynamics and vegetation changes [30]. In the context of climate warming, the trends in moss cover are complex, with few studies showing clear decreases or increases, and the majority of studies indicating neutral responses in Arctic tundra with warmer climates [30]. However, there is little information relating climate trends with shade-tolerant mosses under expanding tundra shrubs. Only two studies have shown an overall decrease in bryophytes with long-term warming or absence of grazing [6, 31], but bryophyte responses were species-specific and did not allow for generalization to shade-tolerant mosses. Nevertheless, current theory predicts that the boreal biome will move into northern latitudinal and elevational tundra [1, 32]. Following these predictions, and accounting for the successional strategies of mosses colonizing suitable habitats [27, 33], it is possible that shade-tolerant moss species can expand with shrub species [34], although local conditions will likely influence their establishment and expansion [6, 30, 35].

**Fig 1:**
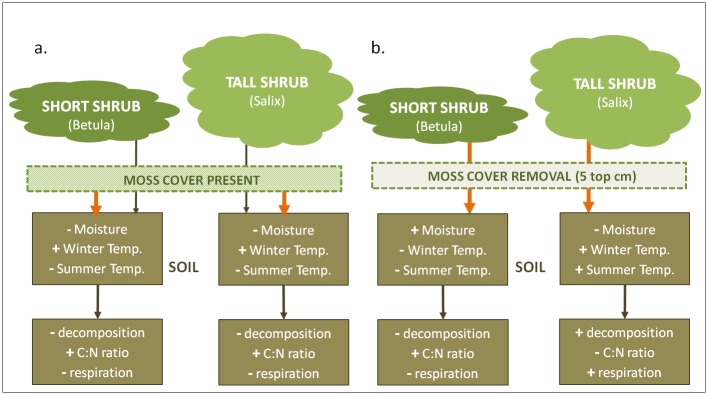
Predicted responses of soil parameters under two dominant expanding shrubs in North American alpine tundra (*Betula* and *Salix*), when moss cover is intact (A) and artificially removed (B). Because of the insulating properties of moss, we hypothesized that when moss cover is intact (A) the effects of mosses will dominate (orange arrows), leading to similar responses in soils irrespective of shrub identity. However, when the active layer of mosses is removed (B), we expected differences in shrub canopy properties to drive soil responses: taller and sparser canopies (*Salix*) will ameliorate conditions for soil decomposition processes, while dense canopies (*Betula*) will maintain cooler soils in summer, slowing down soil processes.

Given this larger context of shrub expansion into alpine and Arctic tundra and the known effect of mosses modulating soil properties and processes in boreal and High Arctic ecosystems, our aim here is to analyze the role of moss cover in mediating soil-shrub interactions in alpine tundra. We conducted a field experiment to determine if the removal of shade-tolerant moss cover affects soil properties and processes under two shrubs that are expanding in North American alpine tundra [4, 36]: *Salix planifolia pulchra* and *Betula glandulosa-nana* complex (hereafter *Salix* and *Betula)*. We predicted that mosses would control the soil environment, either by modulating the effect of the above-moss elements (snow and litter), or by having a direct effect on soil conditions and nutrients. Given the insulating properties of moss, we predicted that moss-intact control plots would have lower summer soil temperature and moisture content, and higher C:N ratios, with subsequent lower decomposition and soil respiration rates, relative to plots where moss had been removed (Fig 1A). In contrast, in the absence of an active moss layer, we predicted that shrub canopy structure (*Salix* vs. *Betula*) would drive the responses of soils; under taller, sparser canopies *(Salix)*. Without the active moss layer, under taller, sparser canopies *(Salix)* we predicted higher summer soil temperatures and soil moisture, leading to increased soil N availability (lower C:N ratio) with higher litter decomposition and soil respiration rates, while the opposite was expected under shorter, denser canopies (*Betula*) in the absence of active moss cover (Fig 1B). To our knowledge, the first attempt to explicitly address the interactive effects of mosses and shrubs on alpine tundra soils under field conditions.

## Methods

### Study area and experimental design

The study was conducted in an alpine valley of the Ruby Range (61°21’N, 138°28’W), SW Yukon, Canada, with summit ridges at around 2,000 m.a.s.l. Treeline and shrubline occur at approximately 1,300 and 1,600 m.a.s.l, respectively. The valley bottom is dominated by shrub tundra (S1 in Walker et al. 2005) with *Salix planifolia pulchra* (*Salix*) and *Betula glandulosa-nana* complex (*Betula*), with shade-tolerant mosses growing underneath. Moderate increases in shrub cover over the last decades have been reported for the study area, with measured rates of increased shrub cover estimated to be ~5 ± 1% per decade [4, 37]. *Salix* spp. and *Betula* spp. are the main genera of shrubs encroaching into tundra in North America in response to warming [4]. The study site is mainly composed by granitic and metamorphic rocks. Soils are poorly developed and show a thin layer of mineral soil and a thicker layer of organic matter from litter accumulation and slow decomposition of tundra vegetation. The study site is located within the traditional territory of the Kluane First Nation who allowed us to conduct ecological research at this site. This land is not privately owned or protected, and no species at risk were sampled or permanently disturbed as part of our study. All of the clipped mosses had regrown within 24 months.

In late summer 2012, 10 sites with similar environmental characteristics (Table 1) were selected within shrub tundra at approximately 1,600 m on a gentle West-facing slope. All sites were selected with pairs of co-occurring shrub species (0.5 m apart from one another) sharing a similar thick layer of shade-tolerant mosses growing underneath (average (±SE) moss depth, including moss litter, 22.43 ±1.11 cm). Although the exact moss canopy structure of the moss layer varied under each shrub, we selected sites without clear visual differences in their composition, thickness or shrub aerial cover (Table 1). The depth of the green moss layer ranged approximately from 4–6 cm and was dominated by six moss species including pleurocarpous (*Hylocomium splendens* and *Rhytidium rugosum)*, and acrocarpous (*Polytrichum juniperinum*, *P*. *strictum*, *Aulacomnium palustre* and *A*.*turgidum*). Other moss species present in the study area, but less abundant, were pleurocarpous (*Saninonia uncinata*, *Tomenthypnum nitens*, *Abietinella abietina and Brachythecium coruscum*) and acrocarpous (*Niphotrichum canescens*, *Dicranum acutifolium*, *D*. *groenlandicum and D*. *elongatum)*. The dominant moss species, present in all plots *(Hylocomium splendens*, *Aulacomnium* and *Polytrichum spp)*, are known to fix atmospheric N through their symbiotic cyanobacterial partners [38, 39].

**Table 1:** Characteristics of the plots in relation to the two shrub species, *Betula* and *Salix*.

	*Betula*	*Salix*	t-value	df	p-value
**Moss cover (%)**	**58.5 ±10.54**	**79.5 ±7.76**	**-2.375**	**9**	**0.041**
Moss depth (cm)	20.38 ±1.69	24.48 ±1.18	-1.911	9	0.088
Shrub areal cover (m^2^)	2.73 ±0.35	2.40 ±0.27	0.707	9	0.497
**Shrub height (m)**	**0.34 ±0.03**	**0.43 ±0.02**	**-2.368**	**9**	**0.042**
Average snow depth (cm)*	16.7 ±1.60	21.5 ±2.42	1.229	5	0.318
Snow duration (days) *	238.4 ±4.45	233.4 ±4.79	0.844	5	0.385
**Proportion PAR** **	**0.30 ±0.17**	**0.46 ±0.19**	**31.868**	**29**	**0.000**

Means ±SE are shown for each shrub species. Differences between plots under each shrub species were tested using paired t tests; t values, degrees of freedom and p values are indicated here.

* For average snow depth and duration, t-values represent F-values from one way ANOVA.

** For the proportion of photosynthetic active radiation reaching the plot surface (proportion PAR), t values represent F-values from LMM with plot within site as a nested random factor.

Significant differences (p<0.05) are indicated in bold.

At each site, four 50x50 cm plots were established; two under each focal shrub species (Table 1). The two plots under each shrub (10 cm apart) were randomly assigned to an experimental manipulation of moss cover (moss removal or unmanipulated control; 10 replicates per shrub species and moss manipulation treatment; S1 Fig). Moss removal consisted of carefully clipping the upper 5 cm of the moss layer, which corresponds to the green photosynthetic active biomass, where most symbiotic cyanobacteria are present and nitrogen fixation rates are highest [25, 38]. By removing this layer could control for the active physiological effects, such as photosynthesis or fixation of atmospheric N, as well as its physical properties, while keeping soil undisturbed. We observed some greening and incipient re-growth of the deeper moss layer in the removal plots at the end of the experiment. Moss removal was conducted at the beginning of the experiment in late summer 2012; measurements were taken during winter 2012 (from Aug 20, 2012 to June 16, 2013) and summer 2013 (from Jun 16 to Aug 18, 2013). To evaluate the effects of removal of shade-tolerant mosses under shrubs with different canopy structures we characterized the snow profile (depth of snowpack and duration of snow cover), assessed light penetration through the shrub canopies, and measured soil properties (summer and winter temperature, moisture and C, N contents) and processes (litter decomposition and soil respiration).

### Effects of canopies: shrub and moss cover, snow profile and light penetration

To account for the main characteristics of the shrub cover and moss cover, we measured several traits; shrub height and aerial shrub cover were obtained by measuring the height and the two main perpendicular axis to the area covered by the immediate shrub individual above the plots. Under the shrubs, moss cover was visually estimated using a 50 by 50 cm frame in each plot, while moss depth was determined by inserting a calibrated metal pin (60 cm long) in the ground before extracting the soil samples.

To characterize the snow profile during winter for each shrub species, 10 snow stakes were installed in Aug. 2012, one per site in the removal plots, five under *Salix* and five under *Betula*. Wooden stakes 1.8 m tall had small temperature loggers (±1°C, Thermochron^®^ iButton^®^–Model DS1921G, Dallas Semiconductor Corporation, USA) attached at 0, 5, 10, 30 and 60 cm above ground; this system ensures a detailed description of the snow profile along the snow-covered season [40, 41]. iButton temperature was recorded six times per day during winter 2012. Air temperature, liquid precipitation and snow depth were simultaneously recorded throughout the year at a meteorological station (1,635 m.a.s.l; Campbell Scientific HPM45c, TE525M tipping bucket, and SR50 sonic ranger; Campbell Scientific Inc., Logan Utah, USA) located 200 m southeast of our experimental plots. Snow depth was determined by the pattern of the diurnal temperature trend recorded by the iButtons, in relation to the air temperature trend, when covered by snow. Average snow depth for each shrub species during winter and duration of snow cover were calculated as the number of days when snow was detected at 0 cm [41].

Shrub canopy structure can affect the penetration of light to the soil. We measured the proportion of photosynthetic active radiation (PAR) reaching the understory under the two species of shrubs in all plots on three occasions during the growing season (16 Jun, 7 Jul, 2 Aug 2013). PAR was measured within a few seconds above and below shrub canopy with a handheld PAR meter (Spectrum Technologies Inc., Model LQM50-6; Apogee Instruments Inc., Logan, Utah, USA). Measurements were repeated four times in each visit and the proportion of PAR reaching the understory was calculated as the ratio of above and below canopy PAR measures.

### Soil properties: temperature, moisture, and nutrient contents

Soil temperature in each of the experimental plots was measured using a temperature sensor (±0.2°C, Onset^®^ HOBO^®^ Micro Station, S-TMB-M006 temperature sensors, Onset, USA) 5 cm below the surface of removal and control plots, at 30 min intervals. To avoid the strong temporal correlation of temperature data at correlative hours, only noon (UTC/GTM -7 h) temperatures were used in the analyses.

Soil volumetric water content (VWC) was measured in the top 12 cm of the surface with a handheld soil probe (Hydrosense; Campbell Scientific, Logan, UT, USA), three times during the summer 2013 at all experimental plots in the same dates as the PAR measurements.

Soil samples (200 cm^3^, 10 cm deep core) were collected in all experimental plots below the moss cover and at the end of summer 2013 in order to compare C and N status under the different treatments. Soil samples were air dried in the field, then oven-dried (60°C 72 h), sieved at 2 mm and finely ground and analyzed individually for total C and N with an Elemental Analyzer (CE-440m Exeter Analytical, UK). N and C content, and C:N ratios were calculated for each plot.

### Soil processes: decomposition rates and soil respiration

To evaluate biological activity in the soil, 80 litter bags were incubated in the experimental plots, 40 during winter and 40 during summer. Litter samples (winter and summer) consisted of senesced leaf material collected from under nearby *Salix* individuals in August 2012, mixed, and used as a standard leaf substrate. After air drying for three days and mixing, litter was subsampled and 2 g placed into 2-mm mesh bags, 10x10 cm in size. Litter bags were buried 5 cm deep in the moss litter layer of all experimental plots (including the removal plots after the removal treatment, so litter bags were always 5 cm deep in the moss layer), and retrieved at the end of each season. Contents of the litter bags were oven-dried (60°C, 72 h) and weighed to the nearest mg. Samples of ‘fresh’ litter from the winter and summer collection (40, 1 g samples each) were oven-dried (60°C, 72 h) and weighed, thus allowing a correction factor to be calculated for initial litter water content, so that dry weights before and after incubation in the field could be compared. Initial litter water content (average percent (±SE), winter samples = 4.3 ±0.57%; summer samples = 4.6 ±0.75%) was subtracted from the initial litter mass placed in the litter bags. Decomposition rates for each period (winter or summer) were calculated as the difference between the initial corrected mass and the final mass after incubation, divided by the number of days elapsed.

Soil respiration (including the moss layer in the control plots) was measured beneath the shrubs in the experimental plots at the end of summer 2013 (1–2 August) using a Li-6400-09 soil CO_2_ flux chamber and Li-COR Li-6400XT portable photosynthesis system (Li-COR Inc., Lincoln NE, USA). PVC soil collars 6.5 cm high were installed in the plots 24 hrs before the soil CO_2_ efflux measurements. Insertion depth (around 5.5 cm into the moss litter) of the soil chamber was recorded to calculate air volume in the chamber; 3 cycles were repeatedly performed at each location on a single visit to get stable measurements, setting ambient CO_2_ concentration as the target.

### Statistical analyses

To compare plot characteristics (moss cover and depth, shrub areal cover and shrub height; Table 1) under *Betula* and *Salix* we used paired t-tests. We used Linear Models (LM) to analyze the effect of shrub identity on snow depth and duration, as we had one observation (snow-stake) per site. For the proportion of PAR reaching the plot surface (mainly moss layer), where we had several measurements per plot during the summer season, we used Linear Mixed Effects Models (LMM) including plot identity nested within site as a random factor.

To evaluate the effects of shrub canopy and moss removal on soil properties and processes we built Linear Mixed Models (LMM). Site was included as a random factor in all cases, except where several measurements were taken in each experimental plot (soil respiration and soil moisture) when plot identity nested within site was included as a random factor. For soil moisture, as several measurements were taken at three times along the season, an additional crossed random factor ‘date’ was included to account for temporal variation. All models included the interaction between shrub species and moss manipulation as a fixed effect variable. In the case of litter decomposition and soil temperature separate models were built for winter and summer.

Modelling assumptions were checked by visually inspecting residual patterns [42]. Whenever model assumptions were not met *a priori* (winter and summer soil temperatures and soil moisture), log transformation of the response variable were enough to correct deviations from normality and homoscedasticity in the model residuals. All analyses were conducted in R 3.1.1 [43] and the package *nlme* for building LMMs.

## Results

### Differences in snow profiles and light penetration between shrub species

**Fig 2:**
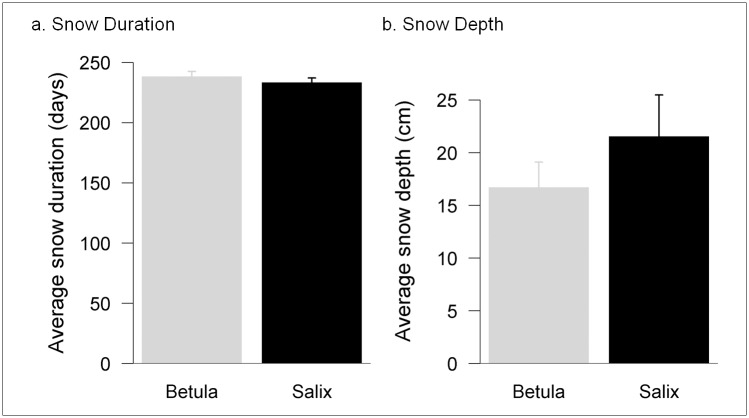
Snow measurements in the experimental plots during winter 2012. Snow depth (cm; **a**) and snow cover duration (days; **b**) values (mean ± SE), during winter 2012 under the two focal shrub species (*Betula* and *Salix*).

*Salix* shrubs were on average taller than *Betula* shrubs (Table 1), but snow profiles (average snow depth and snow duration) did not significantly differ for the two species (Table 1; Fig 2a). Snow cover duration associated with both species was similar (Fig 2b). The proportion of PAR reaching the moss surface was significantly lower under *Betula* compared with *Salix* canopies (Table 1), indicating that *Betula* canopies reflect more sunlight relative to *Salix* canopies [36].

### Soil properties: soil temperature, moisture and nutrients

**Fig 3:**
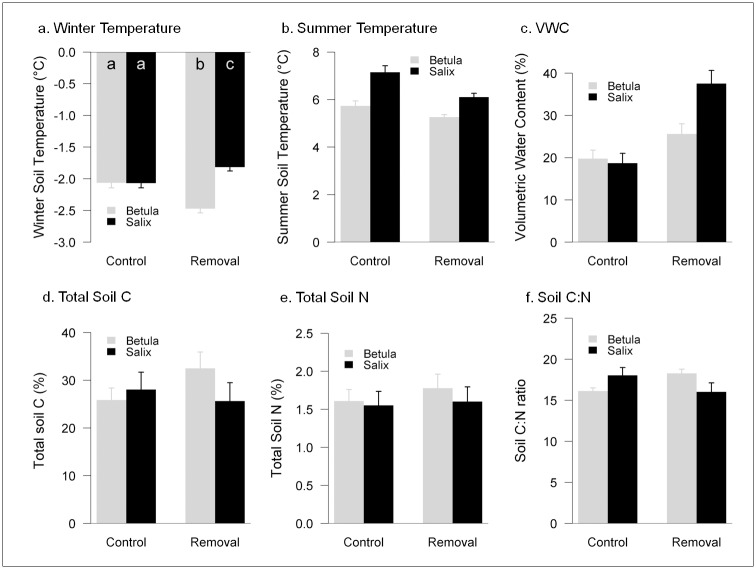
Measurements of soil properties in the experimental plots. Soil properties (winter soil temperature (°C; **a**), summer soil temperature (°C; **b**), soil moisture (volumetric water content; **c**), total soil C (%; **d**), total soil N (%; **e**), and soil C:N ratio (**f**)) of plots with an intact moss layer (control, light grey bars) or with moss experimentally removed (removal, dark grey bars) under *Betula* and *Salix* shrubs. Means and SE are shown; small-case letters indicate statistically significant differences between treatments (shrub identity and moss removal combinations).

Winter soil temperatures were similar under the two shrub species in control plots but differed in moss removal plots (Table 2), with *Salix* having the warmest winter temperatures (Fig 3a). Average (±SE) winter soil temperatures were higher in *Salix* (-1.93 ±0.07°C) than in *Betula* plots (-2.26 ±0.08°C). Moss removal treatment increased temperature differences during winter; moss removal had a warming effect under *Salix* (removal plots were 0.41°C warmer on average than control; Fig 3a) and a cooling effect under *Betula* (0.25°C cooler on average than control; Fig 3a). In contrast, summer soil temperatures showed only significant differences between *Salix* and *Betula* (Table 2; Fig 3b) and there was no effect of moss removal. *Betula* plots had lower summer soil temperatures (5.48 ±0.08°C) compared with *Salix* plots (6.59 ±0.11°C).

**Table 2:** Effects of moss treatment (control or moss removal) and the identity of the overstory shrub species (*Betula* or *Salix*) on soil properties and soil processes derived from Linear Mixed Models (LMM).

**SOIL PROPERTIES**				
**Soil temperature**		**Estimate (±SE)**	**t-value**	**p-value**
Winter (log)	Moss removal	-0.034 (±0.005)	-7.290	0.000
	Shrub species	0.014 (±0.005)	3.039	0.002
	Moss*Shrub	**-0.066 (±0.007)**	**9.971**	**0.000**
Summer (log)	Moss removal	0.014 (±0.033)	0.435	0.664
	**Shrub species**	**0.201 (±0.033)**	**6.107**	**0.000**
**Soil moisture (log)**				
	**Moss removal**	**0.494 (±0.159)**	**3.117**	**0.004**
	Shrub species	0.157 (±0.159)	0.992	0.330
**Soil nutrients**				
Carbon	Moss removal	2.128 (±3.107)	0.685	0.499
	Shrub species	-2.326 (±3.107)	-0.749	0.460
Nitrogen	Moss removal	0.110 (±0.153)	0.718	0.479
	Shrub species	-0.117 (±0.153)	-0.763	0.452
C:N ratio	Moss removal	2.151 (±1.118)	1.924	0.065
	Shrub species	1.920 (±1.118)	1.717	0.098
	Moss*Shrub	**-4.157 (±1.582)**	**-2.628**	**0.014**
**SOIL PROCESSES**				
**Litter decomposition**		**Estimate (±SE)**	**t-value**	**p-value**
Winter	Moss removal	-0.020 (±0.040)	-0.493	0.626
	Shrub species	-0.045 (±0.040)	-1.131	0.268
Summer	Moss removal	-0.257 (±0.360)	-0.715	0.480
	Shrub species	0.500 (±0.360)	1.389	0.176
**Soil respiration**				
	Moss removal	-0.690 (±0.485)	-1.424	0.166
	**Shrub species**	**1.067 (±0.485)**	**2.200**	**0.036**

Log transformation of the response variable to fit model assumptions is indicated in brackets. Significant differences (p<0.05) are indicated in bold.

Differences in soil moisture in summer 2013 were largely driven by moss manipulations, with higher soil moisture in the moss removal plots (32 ±22%) compared with the unmanipulated controls (19 ±17%; Table 2). There were no differences in soil moisture between shrub species (Table 2), but volumetric water content tended to be higher under *Salix* (28 ±23%) than under *Betula* (23 ±17%; Fig 3c).

Neither C nor N content differed between shrub species, the experimental removal of moss, or their interaction (Fig 3d and 3e; Table 1; shrub*moss, LMM; carbon: t = -1.492, p = 0.147; nitrogen: t = -0.120, p = 0.703). However, for soil C:N ratios there was an interactive effect of shrub species identity and moss treatment (Fig 3f): *Betula* moss removal plots had higher C:N ratios than controls, while under *Salix* no differences were observed.

### Soil processes: decomposition rates and soil respiration

**Fig 4:**
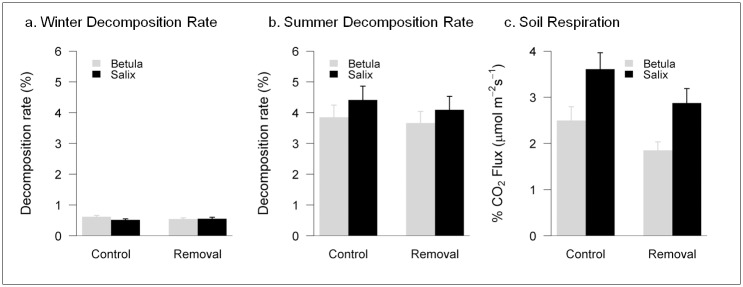
Measurements of soil processes in the experimental plots. Soil processes (winter litter decomposition (mg day^-1^; **a**), summer litter decomposition (mg day^-1^; **b**) and soil respiration (μmol CO_2_ m^-2^s^-1^; **c**), in plots with an intact moss layer (control, light grey bars) or with moss experimentally removed (removal, dark grey bars) under *Betula* and *Salix* shrubs. Means and SE are shown.

Litter decomposition rates were eight times higher in summer than in winter (summer: 4.00±1.30 mg day^-1^; winter: 0.56±0.12 mg day^-1^). Neither shrub species identity, moss manipulation, nor their interaction (LMM, shrub*moss; summer: t = -0.186, p = 0.854; winter: t = 1.453, p = 0.158) had a significant effect on litter decomposition rates in summer or in winter (Table 2; Fig 4a and 4b).

Soil respiration at the end of summer 2013 was higher under *Salix* canopies (3.24±1.86 μmol CO_2_ m^-2^ s^-1^) than under *Betula* (2.17±1.38 μmol CO_2_ m^-2^ s^-1^; t = 2.200, p = 0.036; Table 2, Fig 4c). Although non-significantly, respiration tended to be higher in control plots than in plots where moss had been removed, and this trend was consistent for both shrub species (i.e., non-significant interaction, shrub*moss, t = 0.087, p = 0.931).

## Discussion

The growth and expansion of shrubs (shrubification) is occurring in many subarctic and alpine tundra communities [4, 10, 44, 45]. Current hypotheses for the mechanisms driving this expansion are focused on shrub properties and functional traits, either in their ability to trap snow [9–12, 46] or in the quality and quantity of their litter, which may lead to positive feedback to shrub growth and expansion [17]. Less attention has been devoted to incorporating other factors that may modulate the ecosystem responses to changes. In this study we analyze the role of mosses in key ecosystem properties (soil temperature, moisture and nutrients) and processes (litter decomposition and soil respiration). We hypothesized a regulating effect of mosses, following their direct influence in the dynamics and current changes in boreal and tundra vegetation [30] that would differ under the different shrub species. We observed that mosses modulate or even override the effects of shrubs on soils, by reducing the differences in soil moisture and soil temperatures in summer and winter respectively, and by influencing soil carbon to nitrogen ratios across shrub species.

The presence of mosses reduced soil moisture irrespective of shrub identity. Previous studies have shown that moss cover can either decrease soil moisture by preventing excessive water infiltration into the soil [22, 47], or increase it by avoiding water loss to evapotranspiration [21, 24]. In our study area, the average air temperature during the summer was 9.6°C (minimum 0.4°C, maximum 20.3°C). The total summer precipitation in 2013 was 115.9 mm in 59 distinct events (recorded as hourly totals of which 24 recorded 1 mm or greater at the on-site weather station). Thus, the relatively abundant precipitation evenly distributed during through the summer and combined with cool summer temperatures, may have led to frequent water saturation of the moss cover. Water saturation of the moss layer may actively impede water infiltration, exerting a strong control over soil water balance and its interactions with aboveground vegetation.

Soil temperatures in winter were modulated by moss. Plots with an intact moss cover had similar soil winter temperatures for both shrub species but, when mosses were removed, soil temperatures were significantly lower under *Betula* and higher under *Salix*. The different responses of soil temperatures to the presence of mosses under the two species of shrubs are difficult to explain with the data available, but might be related to variables not measured in the current study, such as snowpack properties [48] or moisture content of mosses which affects thermal insulation of the moss layer [49], or to differences in properties of the moss litter remaining in the experimental plots after experimental removal of the active layer. In this study differences in soil moisture or winter temperature cannot be directly related with snowpack thickness, as we observed no significant differences in snow depth between plots associated with taller *Salix* and shorter *Betula* shrubs. Similarly, no differences were observed between the shrub species in the annual duration of the snow cover. Snow depth measurements were consistent with instrumental records from the adjacent meteorological station (average 0.15–0.20 m throughout the winter), indicative of numerous wind scouring events that keep the snow pack relatively shallow. In our study area snow depth can be highly variable among years, but the strong prevailing winds homogenize the distribution of snow and differences between shrub species might not be as evident [11, 50].

During summer, differences in soil temperature were mainly driven by shrub species identity, with soils in *Betula* plots being on average cooler than those in *Salix* plots. *Betula* has denser, more reflective canopies [36] that allow less light penetration to the understory (Table 1). Increased summer soil temperatures and PAR are consistent with enhanced soil respiration rates under *Salix* relative to *Betula*. The moss removal treatment had minimal effect on summer soil temperatures and soil respiration rates, but both variables tended to be lower in plots where the moss layer was removed. This trend can be explained by the missing active photosynthetic layer of moss, which significantly contributes to ecosystem respiration rates. For example, mosses in Arctic tussock tundra contribute 8.5% of the ecosystem respiration [51]. The effect of moss cover on soil respiration was largely overridden by shrub identity, with higher respiration rates under *Salix*. In our study area *Salix* plots had higher moss cover than *Betula*, so we cannot unambiguously separate the effects of moss cover, the composition of the remaining moss litter layer, and shrub identity on soil respiration. However, recent studies have demonstrated that roots of *Salix* had higher respiration rates than those of *Betula* [51].

We found that C:N ratios were higher when the active moss layer was removed compared to the intact moss under *Betula*, but not under *Salix*. Mosses are able to fix C from the soil and N from and the atmosphere, through their association with cyanobacteria [28]. Mosses also release organic carbon in the form of recalcitrant litter and inorganic N after disturbance events, such as fire or drying and rewetting events [30, 52]. Moss layer depth may also control the availability of nitrate in Arctic tundra, with less mineralization in deeper, cooler and wetter soil environments under moss layers [22, 23]. Our results suggest an interaction with the overstory vegetation, where more open canopy structures (*Salix*) allow more radiation to reach the soil, increasing soil temperature and related nutrient cycling processes. As well, C content of leaf litter seems to be higher in *Betula* than *Salix* [12], which along with cooler soil environments can lead to organic C accumulation in the soil. These differences in the understory soil conditions of the shrub species, with cooler temperatures and higher C litter inputs in *Betula* plots without the active moss layer and its potential N contribution, can lead to the observed increase in CN ratio.

Most key soil properties and processes measured in our study were significantly affected by the removal of the moss active layer (soil moisture), the overstory shrub species (summer soil temperature and soil respiration), or both (winter soil temperature and C:N ratios). Other processes including litter decomposition in winter and summer, were not affected, suggesting a role of both mosses and shrubs in controlling soil nutrient cycles, soil conditions and soil resource availability in tundra soils. By influencing rates of soil respiration, decomposition and mineralization, mosses may affect the heterogeneous responses of shrub expansion. These influences were evident over a single year, and are likely to be cumulative over longer periods with potentially large consequences for tundra communities.

## Supporting Information

S1 Fig Experimental design. In each of 10 experimental sites, four 50 x 50 cm plots were set by pairs under two neighbouring individuals of *Betula glandulosa-nana* complex and *Salix planifolia pulchra*, and each plot in a pair was randomly assigned a moss removal/control treatment. Soil temperatures were monitored for the duration of the study using temperature loggers 5 cm below the surface. One snow stake was set at each site (10 snow stakes total), five located in *Betula moss-removal* (blue stake) and 5 in *Salix moss-removal* plots (red stake). Litter bags were located in each plot during winter 2012–2013 to assess winter decomposition rates, and again in summer 2013 to assess summer decomposition rates. Soil respiration was measured in each plot at the end of summer 2013; collars were installed 24h before respiration measurements were taken, to avoid biases in other measurements derived from soil disturbances.
(TIF)

S1 File Data used in the experiment. The file "Data file S1.xlsx" contains all data used in the analyses of this work. It is divided in 6 data sheets for each of the analyses: "shub_moss" with the information about shrub heights, volume and moss cover per plot; "microhabitat" describes soil moisture (water volumetric content), PAR and moss depths for all plots; "snow_data" describes the snow depths and snow cover duration for all sites; "Soil nutrient" describes the soil C%, N% & CN ratios for all plots; "soil respiration" describes the soil CO_2_ efflux and "litter decomposition" the decomposition rate within the litter bags of winter and summer for all plots.
(XLSX)
