# Supplementary figures and images for: Moss Mediates the Influence of Shrub Species on Soil Properties and Processes in Alpine Tundra
Source: PLoS One. 2016 Oct 19;11(10):e0164143. doi: 10.1371/journal.pone.0164143 (PMC5070840; doi:10.1371/journal.pone.0164143)

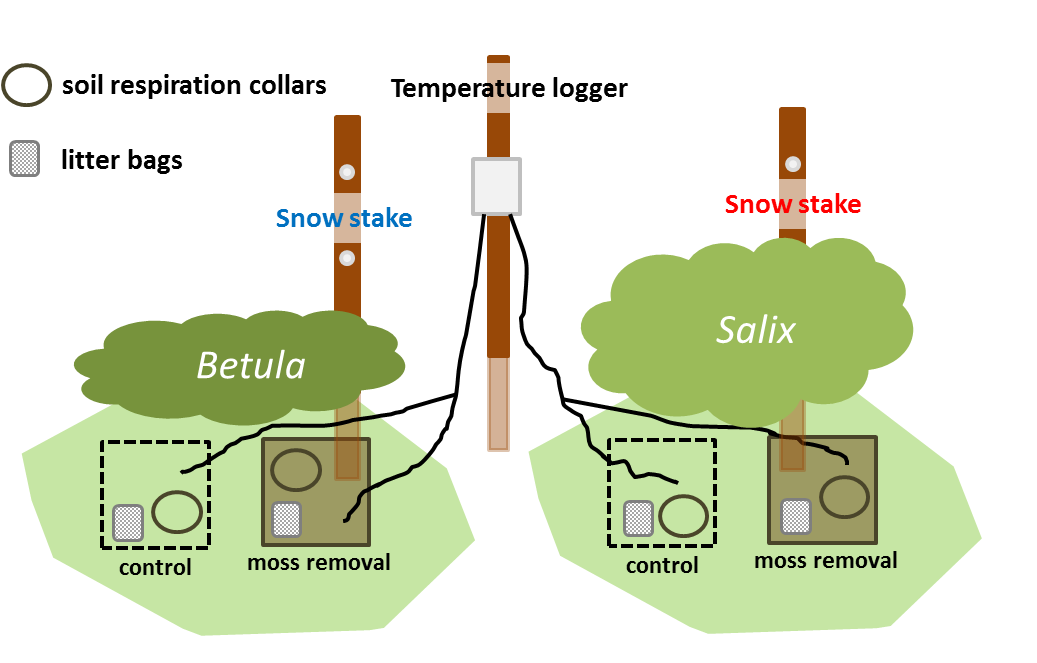

Supplement: S1 Fig — In each of 10 experimental sites, four 50 x 50 cm plots were set by pairs under two neighbouring individuals of Betula glandulosa-nana complex and Salix planifolia pulchra, and each plot in a pair was randomly assigned a moss removal/control treatment. Soil temperatures were monitored for the duration of the study using temperature loggers 5 cm below the surface. One snow stake was set at each site (10 snow stakes total), five located in Betula moss-removal (blue stake) and 5 in Salix moss-removal plots (red stake). Litter bags were located in each plot during winter 2012–2013 to assess winter decomposition rates, and again in summer 2013 to assess summer decomposition rates. Soil respiration was measured in each plot at the end of summer 2013; collars were installed 24h before respiration measurements were taken, to avoid biases in other measurements derived from soil disturbances. (TIF) [file pone.0164143.s001.tif]
